# Embodied Belonging: In/exclusion, Health Care, and Well-Being in a World in Motion

**DOI:** 10.1007/s11013-020-09693-3

**Published:** 2020-11-21

**Authors:** Dominik Mattes, Claudia Lang

**Affiliations:** 1 Collaborative Research Center Affective Societies, Freie Universität Berlin, Berlin, Germany; 2 Institute of Anthropology, University of Leipzig, Leipzig, Germany; 3 Max Planck Institute for Social Anthropology, Halle, Germany

**Keywords:** Belonging, Embodiment, Exclusion, Marginalization, Well-being

## Abstract

In this introduction, we propose the notion of ‘embodied belonging’ as a fruitful analytical heuristic for scholars in medical and psychological anthropology. We envision this notion to help us gain a more nuanced understanding of the entanglements of the political, social, and affective dimensions of belonging and their effects on health, illness, and healing. A focus on embodied belonging, we argue, reveals how displacement, exclusion, and marginalization cause existential and health-related ruptures in people’s lives and bodies, and how affected people, in the struggle for re/emplacement and re/integration, may regain health and sustain their well-being. Covering a variety of regional contexts (Germany/Vietnam, Norway, the UK, Japan), the contributions to this special issue examine how embodied non/belonging is experienced, re/imagined, negotiated, practiced, disrupted, contested, and achieved (or not) by their protagonists, who are excluded and marginalized in diverse ways. Each article highlights the intricate trajectories of how dynamics of non/belonging inscribe themselves in human bodies. They also reveal how belonging can be utilized and drawn on as a forceful means and resource of social resilience, if not (self-)therapy and healing.

## Introduction

The particular moment in history in which we began to write these introductory lines is perhaps just right for intensifying thinking about ‘belonging.’ George Floyd, the 46-year-old African American, who in late May 2020 lost his life in an act of callous police violence in Minneapolis, has just been laid to rest. Sparked by his untimely and appalling death, many countries across the world are witnessing the largest wave of protests against racism and police brutality since the 1960s. Simultaneously, the world finds itself in the midst of the global spread of COVID-19, a ravaging pandemic of unprecedented scale with massive and long-term economic, political, and social consequences for countless individuals and entire societies.

The convergence of these large-scale transformative developments, and each in itself, shine a bright light on the multiple, often fatal, consequences of social exclusion and hierarchized conceptions and experiences of belonging: ever recurring incidents of structural violence against ethnic and other minorities (Basnyat 2017; Bourgois and Schonberg 2009; Farmer 2003); a country’s foreign residents finding themselves stranded in a situation of pandemic emergency due to their non-citizenship (Altay 2020); refugees, asylum seekers, and other marginalized groups struggling for living and housing conditions that would allow them to abide by governmental directives and protect their own health and that of others (Betscher 2020; Schwartz et al. 2020); and the disproportionately high risk for indigenous populations to catch and die from COVID-19 due to their longstanding politically induced structural vulnerabilities (Prates 2020). These are only a few examples that starkly bring to our attention how intimately health, well-being, and the delivery of adequate and equitable health care are interwoven with questions of in/exclusion and non/belonging.

In this introductory article, we wish to theoretically attend to this nexus, a connection that the authors of the present special issue continue to gauge on the basis of their detailed ethnographic observations in diverse geographic and social settings. With one exception (von Poser and Willamowski), the articles of the collection have grown from presentations given at a workshop we convened on behalf of the Work Group Medical Anthropology (German Anthropological Association) at Freie Universität Berlin in 2017 (Meier zu Biesen 2017). In this workshop, we were interested in exploring how non/belonging—understood as both a continuous, dynamic, and often unstable process and a temporarily solidified state of being—affects not only people’s bodily dispositions and constitutions, but also their well-being in a broader sense. We further asked how dynamics of embodied non/belonging shape particular constellations and situations of health care provision, in both enabling and productive, as well as constraining and restrictive, ways.

In exploring health-related transformations in people’s lives caused by processes of de- and reterritorialization, but also by non-geographic forms of social dis- and re-emplacement, we proposed the term ‘embodied belonging’ to analytically connect social, moral, and political-legal aspects of belonging with its affective^1^ and sensorial dimensions. In doing so, we were concerned with how non/belonging matters in the suffering, care, and well-being not only of migrants and refugees, but also of the homeless, disabled persons, and other disadvantaged populations. What are the effects of particular politics of belonging and the corresponding administrative regimes on institutional setups of health care provision, we asked. What are the consequences with regard to people’s capacities to maintain and re-create a sense of belonging and to sustain their well-being? What is the role of religious and other non-biomedical healing practices in achieving and sustaining embodied belonging and well-being? How are the body and the senses entangled in perceptions, disruptions, and re-creations of belonging? And how, finally, are in/capacities to belong and the respective consequences for people’s well-being shaped along fracture lines of social difference such as gender, age, religious affiliation, ethnicity, and legal status?

Aside from this conceptually oriented introduction, the present special issue comprises four ethnography-based articles and is rounded off by a concluding commentary from Sarah Willen. Drawing on their long-term research in settings that range from clinical and non-governmental health care centers to the day-to-day life of un/documented migrants and homeless people, the authors empirically explore health-related processes of in/exclusion and non/belonging in Vietnam/Germany, Norway, the UK, and Japan. They critically engage with particular politics of belonging, as well as corresponding administrative regimes of health care provision, and explicate the detrimental, often literally subcutaneous, consequences for those who are considered not to belong. More than that, they flesh out the bodily facets of specific experiences of deservingness, moral entitlement, and dignity—or the lack thereof—not least by exploring how intimately these are tied to sensorial perceptions of belonging in the interplay with specific material and spatial environments.

It is important to note that the contributions to the present collection do not content themselves with pointing out the obstructive and limiting side of exclusion and non-belonging. They also attend to the productivity and agency involved in marginalized people’s and medical professionals’ strategies, activities, and investments in creating and sustaining spaces of inclusion, participation, mutual recognition, and ultimately care and well-being. It is by placing attention on the construction of such “inhabitable spaces of welcome” (Willen 2019: 18) that we wish to move beyond the mere expression of criticism and offer inspiration for thinking about and transforming medicine and health care into a more equitable and rewarding experience for those in need of care and those who professionally or informally provide it.

In the following section, we will offer a brief overview of what we deem to be the most relevant aspects of recent discussions on belonging in the social sciences, cultural studies, and humanities. In the second section, we show how belonging has been taken up and applied in scholarly works that more specifically relate to the body, health, and well-being, before we offer our specific conceptualization of embodied belonging in the third section. We end with concluding remarks on why we hold this notion to enrich the contemporary medical anthropological analysis, and offer some ways forward in terms of thinking belonging across the boundary between the human and non-human.

## Belonging in the Social Sciences and Humanities

Over the past decade, the notion of belonging has gained increasing attention across various disciplines including anthropology, geography, sociology, psychology, cultural studies, and political science (see Mattes et al. 2019). Often, however, scholars focusing on belonging have failed to state what precisely they meant by the notion, which has prompted some to argue that the concept has been vaguely defined, is ill-theorized, and wields questionable explanatory power (Antonsich 2010; Lähdesmäki et al. 2016). Nonetheless, the notion of belonging apparently bears such significant analytical potential that it keeps scholars from different fields, working on the most diverse questions and phenomena, wrapping their heads around it. As Sarah Wright aptly argues, belonging “seems to have escaped the level of rigorous theorization applied to many other foundational terms” (Wright 2015: 391). And yet, it

is a concept of fundamental importance to people’s lives. Feeling a sense of belonging (or not), being legally, morally or socially recognized as belonging (or not), truly has the power to change lives, to make communities and collectives, to bring together and separate in the most intimate, loving, accepting, exclusionary or violent ways. (ibid.)

In order to benefit from the immense meaningfulness of belonging in scholarly writing, despite the lack of solid theorization, Wright further suggests adopting a “weak theory” approach (cf. Sedgwick 1997; Stewart 2008) to examine “the texture of how it is felt, used, practiced, and lived” and to reflect “on the ways that it is deployed” without “shut[ting] down its multiple meanings and uses” (Wright 2015: 392). She lays out weak theory as a practice that “rather than closing down, categorizing, judging, modelling and getting things ‘right’, […] is open to possibilities, to surprises” (ibid.).

Taking inspiration from this approach, we wish to offer some considerations on belonging, the primary aim of which is not to finally manage what others have not and come up with an exhaustive and definitive delineation of the concept. Instead, we attempt to point out what kind of analytically fruitful moves the notion affords, specifically when it is applied to the study of health and well-being. In other words, we are more concerned with what embodied belonging *does* in terms of the routes it opens up, rather than once and for all pinning down what it *is* and where it ends, and thus foreclosing as yet unseen possibilities to add further aspects to our understanding of it and to increase its relevance for social scientific and humanistic analysis. That being said, let us take a brief look at how belonging has most prominently featured in scholarly debates over the past years.

The first set of works clusters around the *politics of belonging*, which “comprises specific political projects aimed at constructing belonging in particular ways to particular collectivities that are, at the same time, themselves being constructed by these projects in very particular ways” (Yuval-Davis 2006: 197; see also Hall 2013). Without a doubt, such political projects are crucial, in that they not only shape but, significantly, also give rise to phenomena such as nationalism, racism, heteronormativity, and other forms of in/exclusion. Such phenomena forcefully take effect in the lives of people who, caught amidst contemporary ambiguities of globalization, have to cope with ascribed “categories of social location,” which come along with particular “positionalit[ies] along an axis of power” (ibid.: 199, see also Freeman 2007). Thinking about the politics of belonging thus raises questions of citizenship, political rights, and the social entitlements and privileges of individuals and groups vis-à-vis particular political entities (Anthias 2016; Crowley 1999). It is, furthermore, important to always stay alert to the intersectionality involved in such politics, as it is inevitably “inflected by factors like age, gender, class, race, ethnicity, disability, and sexuality. Belonging, whether at the level of the collective or the individual, is never free of dynamics of power” (Mattes et al. 2019: 302).

A second cross-cutting theme that seems particularly relevant to us relates to individuals’ social and moral *embeddedness within social collectives*. To belong, in this sense, is to share a history, values, and practices and, most importantly, to feel recognized and appreciated as a morally equivalent member of a community. Sharing common ground, in this sense, does not only imply the receiving of recognition, appreciation, and other benefits of a material and immaterial kind, but also the readiness to give and contribute to the interests of those who co-constitute a social collective. Often, in fact, belonging is essentially contingent on the continuous and unremitting performance of such commitment, which may be termed the labor of belonging (cf. Grey 2010). In consideration of the conceptual overlaps of belonging and identity, it is important to note that with its emphasis on the “practice and performance of commonality, reciprocity, and mutuality,” the notion of belonging “better address[es] and represent[s] the relationality involved in affective processes of collectivization than the categorical notion of identity” (Mattes et al. 2019: 301; see also Anthias 2013 and Pfaff-Czarnecka 2011).

A third important aspect of the theorizing of belonging as both the process and outcome of affective interrelations concerns *sensation and spatiality*. As Natassia Brenman compellingly argues in her contribution to this special issue, “place can […] be treated as a sociomaterial entity that has powerful potential to affect the social world” (Brenman, this issue). To belong, in the sense of developing spatial attachments, presupposes possibilities of establishing a positive and productive relationship with place. This involves not only the question of whether people feel safe and economically rooted, but also what they sensorially perceive and how they feel within particular spatio-material contexts. Unfamiliar and unwelcoming spatial and material environments can thus lead to affective dissonance between people’s day-to-day sensorial experiences (e.g., concerning smell, sight, and proprioception) and their embodied place-memory, and give rise to feelings of discomfort, estrangement, and disorientation (Wise 2010). Feeling out of place for extended periods of time can, in fact, evoke a profound sense of alienation and disorientation that seriously damages bodies and minds (Lems 2014). But place does not only feature in such daunting and destructive ways. It can also become an important source of resilience and well-being. Engaging in a meaningful relationship with place through particular strategies of material or symbolic (re)appropriation of space, for instance, provides marginalized people with a sense of agency, security, and confidence (cf. Dilger et al. 2018). Actively creating positive affective ties to place through sensorial practices such as food sharing can equally contribute to people’s sense of belonging and well-being (Johnston and Longhurst 2012). Anita von Poser and Edda Willamowski’s contribution to this special issue illustrates well what a powerful therapeutic instrument such practices can become in the treatment of impaired mental health.

As this selective glimpse into different strands of research shows, the concept of belonging is characterized by a remarkable semantic multiplicity. We contend that it is precisely this multiplicity—its capacity to simultaneously bring several, often closely interlocking, dimensions of human existence and experience into view (cf. Anthias 2013; Pfaff-Czarnecka 2011)—that lends the notion its great potential for social scientific analysis. This holds for dynamics of in/exclusion involved in large-scale processes of transformation, such as the current COVID-19 pandemic. But belonging also constitutes a productive conceptual means for the exploration of more unobtrusive yet long-term conditions of social inequality and uneven power relations, which perhaps more subtly, but no less effectually, undermine the health and well-being of particularly categorized groups of people.

## Belonging in Relation to the Body, Health Care, and Well-Being

Scholarship in medical anthropology, the social sciences, and the humanities has shown numerous ways in which non/belonging is intimately linked to health, well-being, and access to health care. What strikes us about these studies is their strong focus on migrants, to the neglect of non-migrant yet equally underprivileged groups such as lower class laborers or homeless and racialized populations. We contend that non-belonging and its health-related effects concern migrants and other marginalized groups in similar—though not always same—ways (see for example Kim this issue), and it is important to think belonging across diverse forms of marginalization.

Aside from this general impression, a first theme that we consider relevant for our considerations on embodied belonging concerns the manifold, often politically induced, structural forms of in/exclusion that impact people’s health and well-being. Building on Farmer’s notion of “structural violence”—i.e., “a host of offensives against human dignity: extreme and relative poverty, social inequalities ranging from racism to gender inequality, and the more spectacular forms of violence that are uncontestedly human rights abuses” (Farmer 2003: 8)—migration scholars have emphasized structural vulnerability. Defined as “a positionality that imposes physical/emotional suffering on specific population groups and individuals in patterned ways” (Quesada, Hart and Bourgois 2011: 440), the term stresses the entanglement of individual ill health and social exclusion (Biehl 2005; Horton 2004; Willen 2012b). Drawing from the idea of access to health care as a fundamental human right (Farmer 2003), structural vulnerability is deeply enmeshed with the politics of belonging. Political exclusion, in the form of illegalization and the denial of citizenship or residence entitlements, deprives individuals of the right to belong (Willen 2012b) or, more generally speaking, of their right to have rights (Arendt 1973). Lacking citizenship and residence rights, unauthorized migrants are often reduced to the status of “bare life” (Agamben 1998), with significant consequences for their well-being. The condition of illegality as a legal-political condition (Bendixsen 2020) often exacerbates migrants’ structural and embodied vulnerability to ill health and suffering and jeopardizes their access to adequate health care. It also exposes them to a state of precarity, and to an impermanent and unstable state of vulnerability (Brenman, this issue). Documented migrants such as first-generation Korean Americans in the US—who are often un/underinsured and thus excluded from health care—similarly face the “double burden of increased health risks from long, stress-laden working hours and lack of access to health care due to the prohibitive costs of health insurance for small business owners” (Kim et al. 2012: 623; see also Horton 2004).

Political exclusion often goes hand in hand with moral exclusion. Undocumented migrants are thus not only excluded from politically defined collectives, but also “from the *moral* community of people whose lives, bodies, illnesses, and injuries are deemed worthy of attention, investment, and concern” (Willen 2012a: 806). In this sense, moral questions of health-related deservingness strongly shape the provision of social and legal entitlements, including access to proper health care. The double exclusion—both political and moral—of undocumented migrants has embodied consequences for their health and well-being, in both experiential and epidemiological terms (Willen 2011). Sarah Horton (2004), for instance, shows how the US public health system differentially constructs Cuban and Mexican immigrants’ deservingness of health care and other benefits associated with citizenship. Hospital administrators implementing Medicaid, she argues, provide care in a way that discerns “deserving” Cuban immigrants from “undeserving” Mexican immigrants. While the former are acknowledged to be responsible and self-disciplined, the latter are denied these decisive neoliberal qualities. In other contexts too, health care providers define specific collectives of migrants as deserving and others as undeserving of proper medical care (Holmes 2006). It is, however, not only health care providers but also migrants themselves who conceptualize their own health-related deservingness. Unauthorized migrants in Israel or the US, for example, do not expect the state to cover the costs of their health care but, to the contrary, are willing to pay these costs on their own (Gonzales and Chavez 2012; Willen 2011). More than that, undeservingness can also become embodied when immigrants, homeless people, or other underprivileged individuals and groups internalize culturalized, racialized, or other stigmatizing stereotypes and discourses of non-belonging (Fassin 2004; Larchanché 2012).

While political and moral exclusion affect health and well-being, particular physical and embodied conditions may also serve as legitimacy for claiming citizenship and the right to equitable and adequate health care. Laurel Bradley (2014), for instance, explored how Filipino caregivers in Israel claim their right to residency, citizenship, and health care based on their “embodied Israeli identity.” They assert this identity by referring to transformed body habits, cultural practices, and ideologies in alignment with Israel’s social norms and political values (ibid.: 261). In this case, belonging becomes part of an expanded understanding of health, inasmuch as the right to health is extended to the right to belong. Medical pathologies or damaged bodies may also become a means for claiming belonging. For example, HIV-positive individuals in Sub-Saharan Africa base claims for belonging and treatment for their disease, vis-à-vis the global community, on their shared therapeutic predicament (Nguyen 2005; Robins 2010). For others, damaged bodies become a means to claim national citizenship or residence (Petryna 2002; Ticktin 2006).

Acquiring belonging through the reproductive body is a second relevant theme that we identify in studies on belonging and health. Larger politics of exclusion from health care, for instance, force undocumented migrant women to sometimes capitalize on biological reproduction to achieve health-related benefits based on a sense of belonging and deservingness as laborers in the host country’s economy. In countries where the law entitles migrant mothers’ babies to citizenship, these babies may also facilitate residency papers and access to health care services for their non-citizen mothers. Kate Goldade (2011) shows how giving birth to children in Costa Rica gives Nicaraguan undocumented migrant women practical benefits, such as access to adequate health care or protection from deportation, as well as symbolic benefits, in terms of facilitating a sense of belonging in the absence of citizenship. Scholars working with migrant mothers in the UK show further ways in which migrant women negotiate and exercise belonging, emplacement, and inhabitance through the reproductive body. Gedalof (2009) delineates how reproduction, including not only childbirth and motherhood but also the reproduction of cultural belonging in the form of dressing, teaching, and caring for their children, gives immigrant mothers a complicated sense of collective belonging in the diaspora. In another study, Qureshi (2014), drawing on studies of place, emplacement, and embodiment, demonstrates the relationship between migration, belonging, and the body that births, but also the centrality of the reproductive body, in terms of how migrant women encounter places and invest them with meaning.

A third important theme is belonging as a source of psychological and social resilience. Many studies at least implicitly suggest a strong correlation between belonging and psychological or social resilience. Belonging fosters resilience as much as resilience facilitates individuals’ and communities’ sense of belonging and their emotional and affective ties with collectives and places. For example, Hermann (2018), in her study on social capital in the face of climate change on an atoll state in the central Pacific, argues that belonging—understood as existential ties to land and people—anchors islanders against the vicissitudes of life and provides them with a resource for facing climate-related challenges. Hermann builds on Hastrup’s notion of social resilience—the “capacity to adapt to stress and change” (Hastrup 2009: 20)—to argue that “resilience in the socio-ecological system, which has now revealed itself to be of planetary scale, resides in people” (ibid.).

In another study, Hudson (2013) focuses on experiences of belonging and well-being among individuals who self-identify as both “mixed race/multiracial and queer” (ibid.: 9). She explores these spaces as “community borderlands” or “spaces that are shifting, polyvocal, and multidimensional; […] [which] embody, transform, and resist systems and cultures of oppression, impacting the material realities and lived lives of their occupants and visitors alike” (ibid.: 3f). Challenging the assumption that liminal status is a source of chronic stress and deteriorated well-being, she demonstrates community borderland experiences as actually enhancing resilience and well-being by providing a sense of connectedness through increased social and material resources, and a sense of safety and community belonging. Similarly, Sliwinska (2015) looks at in-between spaces as ‘edge habitats’ that lead to a greater diversity and permeability of social locations and facilitate women’s experience of multiple embodied belonging at a transnational level. She suggests the term ‘denizenship’—understood as the lived experience of embodied belonging—as a heuristic to grasp the hospitality of spaces that welcome the ‘Other’ and make a person feel at home, as negotiated by both the host and the im/migrant.

Therapeutic relationships themselves can also contribute to a sense of belonging and well-being; this is a fourth relevant theme. For many im/migrants, access to health care services entails a sense of inclusion and many seek out psychiatric help in their search for belonging and restoring a cohesive sense of self. Lindqvist and Wettergren (2018) show how for migrant women in Sweden, the psychotherapeutic encounter becomes a “rewarding relational place where migrants can heal their damaged social bonds and forge new ties” (ibid.: n.p.). It is a space for learning and experimenting with habitual practices of belonging to the host society. For a group of Vietnamese migrants in Berlin, too, joint “go-alongs,” as a form of spatial memory and care outside the clinic, enable a sense of belonging within a context that is otherwise shaped by experiences of non-belonging and social exclusion (von Poser and Willamowski 2020). In Natassia Brenman’s article (this issue), an intercultural psychotherapy center in the UK is designed as a welcoming space and a way for irregular migrants to constitute inclusion and embodied belonging. And for the Japanese homeless people featured in Jieun Kim’s article (this issue), commitment to health care itself becomes a mode of alternative belonging.

This brings us to a final theme: the affective dimension of health and well-being. Building upon the literature on emplacement, embodiment, and belonging (Ahmed 1999; Massumi 2002; Wise 2010) that emphasizes the sensorially mediated affective relations between people, space, and belonging, recent scholarship has demonstrated that “the body is both experienced through place and engages place” (Dyck 2006: 5), and it has explored the effects of these entanglements on health and well-being (Gardner 2002; Unnithan Kumar 2015; Raffaetà and Duff 2013). Places have material effects on people’s lives, feelings, and bodily experiences. Bodies can be in and out of place. Diseased, distressed, or aging bodies play an important role in how places are perceived and acted upon, as these bodies are themselves produced by processes of emplacement. Isabel Dyck, writing about Sikh immigrant women in Canada, suggests that accounts of health and illness are “moral tales about ‘being in place’ constructed about the body at different scales” (Dyck 2006: 5), remaking both place and bodies. Similarly, Unnithan Kumar (2015), focusing on labor migrants in India, demonstrates the intimate interrelations of emplacement—the process through which places are actively sensed and invested with meaning—, birth, motherhood, and belonging. Raffaetà and Duff (2013) explore Ecuadorian migrants’ sense of belonging in Italy as an assemblage of social, material, and affective resonances with place, and demonstrate the effects on their experience of health and well-being. Bexley (2007), finally, explores the politics of belonging for a young generation of East Timorese by focusing on seeing, hearing, and feeling place as an affective and embodied experience of belonging. All of these examples demonstrate how essential place and materiality are for people to develop a sense of both belonging and non-belonging.

As this brief review shows, a considerable amount of research in the social sciences has explored the entanglements of varying kinds of in/exclusion with the body, health, and well-being, even if not necessarily making use of the term belonging. However, the majority of these studies focuses exclusively on the political, the physical/psychological, or the affective, i.e., spatio-sensorial, dimensions of belonging. It is this analytic constriction that we wish to respond to through our specific conceptualization of embodied belonging, with the explicit aim of bringing into view these dimensions’ mutual implications and connectedness.

## Embodied Belonging

We envision that the notion of embodied belonging can help us gain a more nuanced understanding of how the above-mentioned dimensions of belonging play out in the domain of health, illness, and healing. More specifically, while acknowledging that the degree of impact of these dimensions may differ from one situation to the other, we suggest that it is the specific focus on how these dimensions become relevant in people’s lives in *interaction* that enhances the depth of anthropological analysis. Political-economic exclusion and marginalization, for instance, often directly translate into spatio-material living conditions that are highly detrimental for both physical and mental health (cf. Krieger 2020). The different dimensions of exclusion and non-belonging, in other words, can go hand in hand. One can cause the other, they may converge and even reinforce each other, resulting in deleterious spirals of declining health and well-being. Conversely, particular activities and strategies regarding one of these dimensions may be a means to counter the harm being suffered in regard to another. Jieun Kim’s article in this special issue serves as an illustration of this, as it shows how effective the creation and provision of a protected spatio-sensorial medical environment imbued with care and moral concern can be for the health and well-being of otherwise grossly marginalized and disadvantaged unemployed and homeless people in Japan.

This being said, why do we wish to speak about “embodied” belonging? What analytical surplus does it convey in comparison to speaking of belonging without this additional specification? In our view, the qualifier ‘embodied’ is particularly geared toward emphasizing three distinct but often interrelated ways in which particular social conditions relate to and are inscribed in human bodies (cf. Scheper-Hughes and Lock 1987).

First, embodied belonging indicates the biological and ultimately epidemiological consequences of socio-economic and political inequality and exclusion for human health, for instance in the form of “differential disease rates and […] variances in morbidity and mortality between social groups” (Nguyen and Peschard 2003: 447; see also Kim this issue; Krieger 2004; Willen 2012b) and among societies on a global scale. This also includes the unequal health-related consequences of anthropogenic climate change and environmental crisis. With debates around the Anthropocene and the postgenomic era, the environment that humans are constantly transforming contributes to ever-increasing inequalities and intensified misery, which are inscribed on bodies and psyches across generations (Lock 2020). Such an inscription of social and ecological environments into bodies has been productively described with the approach of “environmental epigenetics,” a term that “glosses investigations into the lasting effects of toxic exposures, malnutrition, abuse, social isolation, and other troubling events on the mental health and behavior of individuals and their families” (Lock 2020: 26). In all these cases, bodies literally incorporate and “tell stories” (Krieger 2005) about their social, political, economic, and ecological environments across scales (cf. Willen 2011).

Second, in a phenomenological vein, embodied belonging refers to the impact of restrictive socio-political environments on subjectivities, one’s sense of self, and perceptions of the body (Kim et al. 2012; Willen 2007). Politically, socially, economically, and culturally excluded individuals internalize their non-belonging and exclusion in a process of embodiment that profoundly shapes their behaviors, practices, self-conceptions, and habitus (cf. Quesada et al. 2011). Placing emphasis on this process and drawing on the theoretical frame of critical phenomenology (Desjarlais 1997; Willen 2007), the notion of embodied belonging is intended to link the embodied lived experience of non/belonging as a mode of being-in-the-world to the politics of belonging. For many migrants, this being-in-the-world in the form of non-belonging includes experiencing themselves through the Othering gaze of their host society. The persistence of this Othering gaze upon their bodies renders them beings that Anwen Tormey describes, citing Fanon (1990), as “sealed into crushing objecthood” (Tormey 2007: 81–82). Synnøve Bendixsen (2020) further shows how irregular migrants experiencing stigmatization and social exclusion in Norway, also within contexts of health care, develop a sense of embodied non-belonging that significantly impacts their well-being. Only with the help of friendships and trustful relationships with members of their religious communities do they manage to counter this highly harmful situation and rebuild a comforting sense of belonging.

A third important point about embodied belonging is that it brings into view the impact of the spatio-sensorial qualities of particular locales and situations on people’s sense of belonging and well-being (Cartwright 2007; Lems 2014; Wise 2010). Referring to our understanding of affect (see footnote 1), we highlight that an essential feature of creating, sensing, and sustaining belonging is that these are always processes involving mutually impacting and responding bodies and materialities of different kinds, be they human, non-, other-, or more-than-human. While such fundamental relationality comes with the risk of humans being exposed to numerous unhealthy and harmful environments throughout their lives, it also means that through intentionally and strategically shaping and designing these environments, a sense of belonging—and by implication, health and well-being—may be equally enhanced (Brenman this issue; von Poser and Willamowski 2020).

Based on these considerations, we propose that the notion of embodied belonging constitutes a particularly fruitful analytical heuristic for scholars in medical and psychological anthropology. By bringing to the fore the epidemiological, the phenomenological, and the spatio-sensorial as three closely entangled dimensions, it helps us to better understand how displacement, exclusion, and marginalization cause existential and health-related ruptures in people’s lives and bodies, and how affected people, in the struggle for re/emplacement and re/integration, may regain health and sustain their well-being.

## Chapter Summaries and Concluding Remarks

Covering a variety of regional contexts (Germany/Vietnam, Norway, the UK, Japan), the contributions to this special issue examine how embodied non/belonging is experienced, re/imagined, negotiated, practiced, disrupted, contested, and achieved (or not) by their protagonists, who are subjected to different forms of exclusion and marginalization. The authors precisely carve out the ways in which these processes impact on and are shaped by configurations of health care and concerned persons’ physical, mental, and social constitution. Each article thereby convincingly highlights in its own way the intricate trajectories of how dynamics of non/belonging inscribe themselves in human bodies. They also reveal how belonging can be utilized and drawn on as a forceful means and resource of social resilience, if not (self-)therapy and healing.

Synnøve Bendixsen provides a phenomenological and bodily-aware account of irregular migrants’ lifeworlds in Oslo and Bergen, Norway. Drawing from these migrants’ narratives of their everyday lives, she unveils non-belonging as a consequence of processes of illegalization that constitute irregular migrants as undeserving of care and well-being. Non-belonging here emerges as the embodied effect of a violent mode of governmentality that includes laws, health care structures, and migration management rationalities. The embodied consequences comprise somatic and psychological pain and distress, which are exacerbated by delayed health care, deteriorated health conditions, and self-exclusion in an already exclusionary health care system. Bendixsen does not stop at these deeply embodied experiences of “existential displacement,” however, but goes on to explore irregular migrants’ strategies to reinstitute a sense of belonging that, as she shows, becomes a source of resilience. Relationships in substitute health care services, religious communities, and friendships contribute to the migrants’ meaningful ways of being-in-the-world and, at least partially, rebuilding belonging.

Natassia Brenman addresses precarious belonging in the voluntary sector of mental health care in the UK in times of austerity. Her contribution focuses on an intercultural psychotherapy center in London serving a range of minority ethnic and im/migrant communities. Using a method of visual mapping to access spatialized experiences and the sociomateriality of belonging, Brenman shows how places of care “participate in, act on and enact belonging or non-belonging” (Brenman, this issue). In this context, embodied non/belonging not only emerges as a product of the relations between people, spaces, and materials, but is also revealed to be deeply precarious. The center was created as part of a politics of access that aimed to include the otherwise excluded within the wider health care system. In some cases, this paradoxically leads to a sense among clients of not being vulnerable enough, of not belonging to the particular intended client group and thus not deserving of care. Clients paradoxically feel simultaneously to be in and out of place. In these relations between people, spaces, and materials, belonging emerges as deeply precarious, constantly being made and re-made. Precarious belonging is itself linked to the unstable status of places of care in the voluntary sector, within the broader landscape of care and austerity.

Anita von Poser and Edda Willamowski present the results of some remarkably unorthodox therapeutic techniques that they, as anthropologists, developed together with psychiatrists and psychologists in the context of psychotherapeutic treatment for elderly Vietnamese migrants in Berlin. Through what they term “phenomenological go-alongs,” they gained rich insight into the spatio-sensorial situations and surroundings that led to these migrants’ traumatization during war, displacement, and migration. Importantly, however, this is not where the story ends. Drawing on the patients’ embodied memories of their Vietnamese homes and familiar sensorial environments, which were evoked and collectively discussed during affect-abundant group excursions outside of the clinic, the therapeutic team was further able to foster the emergence of a temporary yet therapeutically relevant “affective community” (Zink 2019). In this community, the group members “experienced a sensual place of mutual belonging,” (von Poser and Willamowski 2020: 610) and this demonstrates the significant potential of sensorially sensitive research and therapeutic methods. In other words, the Vietnamese patients’ embodied knowledge was not only elicited in order to enhance anthropological knowledge production but was, significantly, turned into an effective resource for healing.

Jieun Kim presents an equally impressive and no less encouraging account of how medical, social, and moral concern for the marginalized becomes institutionalized in her article on health care provision in Japanese underclass enclaves. She explores how in one such quarter called *Yoseba* in Yokohama, medical activists established a clinic for day laborers and homeless people who, due to specific societal notions of relationality and care, were socially excluded and considered unworthy of any form of care. The clinic serves as a vivid example of the multilayeredness of embodied belonging and demonstrates how effective it can be to synergetically attend to its many dimensions when it comes to enabling processes of healing and rehabilitation. The clinical staff thus not only made efforts to provide proper medical care to the otherwise neglected. More than that, and just as important for patients’ long-term therapeutic progress, was the creation of a specific spatial environment that made patients feel comfortable and that they belonged, and the practical commitment to, and sensorial engagement with, staff and fellow patients at their own rhythm—for instance, in the form of bringing and sharing tea or food. Establishing mutual moral recognition and meaningful social relations between patients and clinic staff was equally essential for patients’ trajectories of convalescence. These relations did not even come to an end when a patient passed away. Specific forms of “necrosociality,” including particular practices of remembering the deceased within the clinic, further contributed to the remedial “alternative logic of belonging” (Kim, this issue) that significantly contributed to breaking patients’ social isolation and enhancing their perceived self-efficacy.

Across all of the contributions to this special issue, resilient ties to fellow human beings and spatio-material attachments feature as foundational prerequisites for the emergence of embodied belonging and associated processes of social, physical, and mental recovery. What is largely absent in these accounts, however—and we wish to mention this as an inspiration for further research on embodied belonging, rather than as an indication of the articles’ shortcomings—is an analytical outreach toward another kind of relationality, namely concerning people’s intricate relations with ecology, in the sense of particular microbial and viral environments. The work of cultural studies scholar Venla Oikkonen serves as an example of how to expand our thinking when it comes to conceptualizing belonging in relation to health and the body beyond the dimensions that we have attended to above. Such an expansion seems particularly relevant in contexts such as the current COVID-19 pandemic.

In her study of what she calls “partial immunities,” Oikkonen (2018) recapitulates historical scientific research on the development of human antibodies in contexts of viral pandemics and emphasizes the role of “viral memories” (ibid.: 6–8) in the constitution of “immunological communities” (ibid.: 10). With the latter term, she designates collectives of bodies that had been exposed to the same kind of influenza virus in the past and who, on the basis of their shared partial immunity to the pathogen, and without being aware of it, “belong” to an immunological community, or possibly to multiple such communities. Particularly inspiring about this concept is that it significantly extends the matter of belonging beyond the here and now. It clearly “draws attention to how traces of the past are stored in biological and material entities (antibodies, viruses, proteins), and how such material traces engender connections that tie the present to the events and places of the past” (ibid.: 8). This being said, it is important to note that even this unwitting, yet highly health-relevant, form of embodied belonging is shaped by socio-economic inequalities and uneven power relations, rather than being the result of contingent human and microbial mobility. “[I]nfections and immunities are not a matter of choice” (ibid.: 7), Oikkonen reminds us. Rather, their disproportionate distribution is a consequence of “geopolitical power relations, colonial legacies and class structures [that determine] how people may protect themselves against epidemics through sanitation, access to health care, or [the] availability of vaccines and antivirals” (ibid.: 9).

The current COVID-19 pandemic strikingly corroborates this argument, as it relentlessly lays bare different sub-populations’ grossly varying vulnerabilities and exposure to both the virus and the social consequences of the fight against it. Furthermore, in pandemic times, the notion of immunity attains powerful potential for social critique in yet another sense: it forces us to reconsider our assumptions about the relation between microbes and the human body, assumptions that have long shaped our conceptions of the human immune system. Instead of pursuing strict mutual exclusion and boundaries between microbes and the body, the notion compels us to view “humans as fundamentally entangled with other life forms—viruses, bacteria, or other species” (ibid.: 4). This, in turn, poses the challenge to think of other, more productive and possibly healthier, ways of co-existence. Embodied belonging, then, becomes not only a matter of whether or not, how, where, and when we belong. Equally pertinent is the question of who and what belongs to, and forms part of, our bodies.

While these considerations may point to some ways of further complicating the notion of embodied belonging in theoretical terms, the contributions to this special issue powerfully illustrate the profound concrete impact that exclusionary ways of dealing with medical crisis, transnational migration, and socio-economic precarity exert on people’s sense of non/belonging. By emphasizing the relevance of non/belonging for matters of health and well-being, the collection demonstrates the specific contribution that medical and psychological anthropology can make to the analysis of the intimate interplay between contemporary politics and the human body. This, we hope, will be instructive for clinicians, therapists, public health professionals, and policymakers in charge of promoting, organizing, administrating, and practicing multiple forms of healing—a task that is perhaps more daunting and challenging than ever amid the multiple converging crises of the contemporary world.

## Footnotes

1 We understand affects here in a broad sense as “material and ideational relations that […] increase or diminish [human and other actors’] existential capacities in relation to their surroundings and all other actors and entities present in a situation” (Slaby and Mühlhoff 2019: 27).
